# Safety and efficacy of sucroferric oxyhydroxide in pediatric patients with chronic kidney disease

**DOI:** 10.1007/s00467-020-04805-y

**Published:** 2020-10-27

**Authors:** Larry A. Greenbaum, Nikola Jeck, Günter Klaus, Marc Fila, Cristina Stoica, Sahar Fathallah-Shaykh, Ana Paredes, Larysa Wickman, Raoul Nelson, Rita D. Swinford, Carolyn Larkins Abitbol, Mihaela Balgradean, Augustina Jankauskiene, Amandine Perrin, Milica Enoiu, Sun-Young Ahn

**Affiliations:** 1 Division of Pediatric Nephrology, Emory University School of Medicine and Children’s Healthcare of Atlanta, 2015 Uppergate Drive NE, Atlanta, GA 30322 USA; 2 KfH Pediatric Kidney Center and Department of Pediatrics II, Philipps-University, Marburg, Germany; 3 CHU Hôpital Arnaud de Villeneuve, Montpellier, France; 4 Institutul Clinic Fundeni, Bucharest, Romania; 5 University of Alabama at Birmingham, Birmingham, AL USA; 6 Nicklaus Children’s Hospital, Miami, FL USA; 7 C.S Mott Children’s Hospital, Michigan Medicine, Ann Arbor, MI USA; 8 University of Utah, Salt Lake City, UT USA; 9 The University of Texas Medical School at Houston, Houston, TX USA; 10 University of Miami - Miller School of Medicine, Miami, FL USA; 11 UMF ‘Carol Davila’ Spitalul Clinic de Urgenţă pentru copii “Maria Sklodowska Curie”, Bucharest, Romania; 12 Vilnius University hospital Santaros klinikos, Vilnius University, Vilnius, Lithuania; 13 Vifor Pharma Management Ltd., Glattbrugg, Switzerland; 14 Children’s National Hospital, The George Washington University, Washington, DC USA

**Keywords:** Chronic kidney disease, Hyperphosphatemia, Children, Phosphate binder, Sucroferric oxyhydroxide, Safety profile

## Abstract

**Background:**

Pediatric patients with advanced chronic kidney disease (CKD) are often prescribed oral phosphate binders (PBs) for the management of hyperphosphatemia. However, available PBs have limitations, including unfavorable tolerability and safety.

**Methods:**

This phase 3, multicenter, randomized, open-label study investigated safety and efficacy of sucroferric oxyhydroxide (SFOH) in pediatric and adolescent subjects with CKD and hyperphosphatemia. Subjects were randomized to SFOH or calcium acetate (CaAc) for a 10-week dose titration (stage 1), followed by a 24-week safety extension (stage 2). Primary efficacy endpoint was change in serum phosphorus from baseline to the end of stage 1 in the SFOH group. Safety endpoints included treatment-emergent adverse events (TEAEs).

**Results:**

Eighty-five subjects (2–18 years) were randomized and treated (SFOH, *n* = 66; CaAc, *n* = 19). Serum phosphorus reduction from baseline to the end of stage 1 in the overall SFOH group (least squares [LS] mean ± standard error [SE]) was − 0.488 ± 0.186 mg/dL; *p* = 0.011 (post hoc analysis). Significant reductions in serum phosphorus were observed in subjects aged ≥ 12 to ≤ 18 years (LS mean ± SE − 0.460 ± 0.195 mg/dL; *p* = 0.024) and subjects with serum phosphorus above age-related normal ranges at baseline (LS mean ± SE − 0.942 ± 0.246 mg/dL; *p* = 0.005). Similar proportions of subjects reported ≥ 1 TEAE in the SFOH (75.8%) and CaAc (73.7%) groups. Withdrawal due to TEAEs was more common with CaAc (31.6%) than with SFOH (18.2%).

**Conclusions:**

SFOH effectively managed serum phosphorus in pediatric patients with a low pill burden and a safety profile consistent with that reported in adult patients.

**Electronic supplementary material:**

The online version of this article (10.1007/s00467-020-04805-y) contains supplementary material, which is available to authorized users.

## Introduction

Hyperphosphatemia is a common complication in patients with chronic kidney disease (CKD) [1, 2], particularly those with kidney failure, in whom elevated serum phosphorus has been identified as a risk factor for mortality, morbidity, and hospitalizations [3–5]. Hyperphosphatemia contributes to several CKD complications, including secondary hyperparathyroidism [6], mineral and bone disorder (MBD) [7], and development of cardiovascular disease [3, 8]. Optimal management of hyperphosphatemia is particularly important in pediatric CKD patients for prevention of skeletal abnormalities and growth impairment, as well as for preservation of long-term cardiovascular health [9].

Dietary phosphate restriction alone rarely provides adequate serum phosphorus control in advanced CKD, and severe restriction in pediatric patients may adversely impact the patient’s growth and development by limiting protein intake [10]. In addition, the use of dialysis for the removal of excess phosphate is not very effective, as phosphate is mainly stored intracellularly. Many CKD patients, therefore, require treatment with oral phosphate binders (PBs) to inhibit gastrointestinal (GI) absorption of dietary phosphate [6].

PBs are widely prescribed to pediatric patients with advanced CKD, though not all of these agents are approved for this indication. Calcium-based PBs, including calcium acetate and calcium carbonate, have been extensively studied in pediatric CKD [11, 12], but are associated with a risk of hypercalcemia and vascular calcification [13–15]. Non-calcium PB options include sevelamer and lanthanum carbonate. Sevelamer is a non-metallic anion-exchange resin available as a carbonate or hydrochloride salt [16, 17]. Sevelamer carbonate is the only non-calcium-based PB currently approved for use in pediatric CKD patients (6 years and older) [17]. Although sevelamer effectively reduces serum phosphorus levels, it has a relatively high pill burden when taken in tablet form, requires swallowing of large tablets without chewing, and is associated with GI side effects, all of which may contribute to poor compliance. The powder form of sevelamer carbonate also requires a large dose that may decrease compliance. In addition, sevelamer may also reduce the GI absorption of fat-soluble vitamins [16, 17]. Lanthanum carbonate is a potent phosphate binder that provides effective serum phosphorus control, with a low daily pill burden. However, the observed deposition of lanthanum in several organs (e.g., bone, liver) has raised potential toxicity concerns about its use [18–20]. Given the limitations of the available calcium- and non-calcium PB therapies, new effective agents with good safety and tolerability profiles are needed for the treatment of hyperphosphatemia in both adult and pediatric CKD patients.

Sucroferric oxyhydroxide (SFOH) is an iron-based PB with a low pill burden indicated for the treatment of hyperphosphatemia in adult dialysis patients. Clinical studies in adults have demonstrated that SFOH is well tolerated and provides effective long-term serum phosphorus control [21–23]. However, there are no data relating to its safety and efficacy in children with CKD.

The aim of this clinical study was to investigate the safety and efficacy of SFOH in pediatric and adolescent patients with CKD and to provide dosing information for this patient population.

## Methods

### Study design

**Fig. 1:**
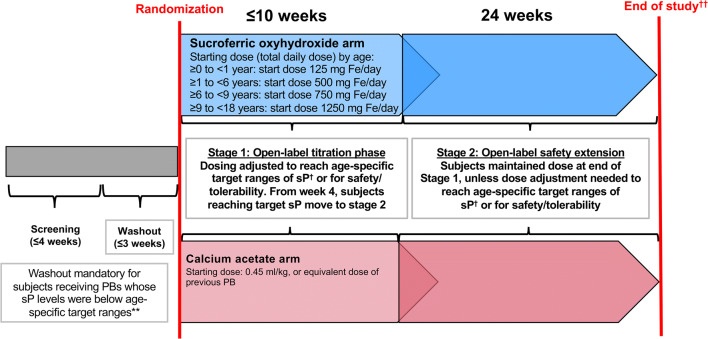
Study design. **Age-related sP targets for washout period 0 to < 6 months, > 8.1 mg/dL; ≥ 6 months to < 1 year, > 7.1 mg/dL; ≥ 1 year to < 6 years, > 6.3 mg/dL; ≥ 6 years to < 13 years, > 5.5 mg/dL; ≥ 13 years to < 18 years, > 4.2 mg/dL. ^†^Age-related sP targets post-randomization 0 to 1 year, 5.0–7.8 mg/dL; ≥ 1 year to < 6 years, 4.5–6.5 mg/dL; ≥ 6 years to < 13 years, 3.6–5.8 mg/dL; ≥ 13 years to < 18 years, 2.3–4.5 mg/dL [27]. ^††^All subjects (including withdrawn) followed for 14 days after the last study visit. PBs, phosphate binders; sP, serum phosphorus

This was a phase 3, multicenter, randomized, prospective, open-label, active-controlled trial conducted in 41 centers in 7 countries (24 centers in the United States [US], 17 in Europe). The study design is shown in Fig. 1. Following a screening period of up to 4 weeks, and a washout period of up to 3 weeks (for participants previously taking PBs with a serum phosphorus below the age-specific target range), eligible subjects were randomized to SFOH or calcium acetate (CaAc) for a dose titration period of up to 10 weeks (stage 1), followed by a 24-week safety extension (stage 2). All randomized subjects were followed for 14 days after their last study visit. The study planned to randomize approximately 130 subjects to receive either SFOH (~ 100 subjects) or the active comparator, CaAc (~ 30 subjects). Randomization was stratified by age group (0 to < 1 year, ≥ 1 to < 6 years, ≥ 6 to < 9, and ≥ 9 to < 18 years) and aimed to randomize minimum numbers of subjects per age group. The study (ClinicalTrials.gov: NCT02688764) was approved by the institutional review boards at each participating center, was conducted in accordance with the Declaration of Helsinki principles [24], and was in compliance with the International Council for Harmonization (ICH) E6 Guideline for Good Clinical Practice (GCP) [25], and with regulations applicable in the respective countries. All subjects or their parents/legal guardians provided written informed consent/assent prior to any study-specific procedures. An independent Data and Safety Monitoring Board was constituted to protect the safety of study participants.

### Study participants and assessments

The study enrolled subjects with CKD and hyperphosphatemia defined according to age-specific criteria, as detailed in Fig. 1. Eligible subjects were either PB-naïve or had been receiving stable doses of a maximum of two PBs for at least 1 month prior to screening. Subjects receiving prior PB therapy with serum phosphorus below the age-specific target were required to undergo a washout period before randomization. Subjects already receiving a PB whose serum phosphorus levels met age-specific target levels were eligible for randomization without a washout; prior PBs were discontinued. All subjects were required to have CKD and subjects ≥ 1 year old were required to have stage 4 or 5 CKD defined by an estimated glomerular filtration rate (eGFR) < 30 mL/min/1.73 m^2^ [26], or stage 5D CKD on at least 2 months of adequate maintenance hemodialysis (HD) or peritoneal dialysis (PD) before screening. As far as possible, dialysis regimens were not changed during the study. Concomitant medications that may influence serum phosphorus (e.g., vitamin D, vitamin D analogues and calcimimetics), were, per protocol, not changed as far as possible during the study. Counseling regarding dietary phosphorus per local practice was encouraged.

Exclusion criteria included hypercalcemia (per age-specific definition), hypocalcemia (serum total corrected calcium < 7.6 mg/dL at screening), and intact parathyroid hormone (iPTH) > 700 pg/mL at screening. A full list of exclusion criteria is provided in Supplementary Table 1.

A schedule of study visits and associated assessments is provided in Supplementary Fig. 1. Following randomization, treatment period visits took place at weeks 1, 2, 4, 6, 8, and 10 (stage 1), and at weeks 14, 18, 22, 28, and 34 (stage 2) after the baseline visit. For HD subjects, blood samples were obtained prior to a HD session and study visits were planned after a maximum interdialytic period of 48 h and on the same day and same session each week, as far as possible. Blood samples were analyzed by the central laboratory whenever possible.

## Randomization and study treatments

Eligible subjects were randomized to treatment with SFOH or CaAc at the baseline visit; other PBs were discontinued and prohibited during the study. SFOH was provided in two formulations: a formulated powder for oral suspension (doses corresponding to 125, 250, and 500 mg of iron) in sachet packs for subjects < 6 years old, and chewable tablets (doses corresponding to 250 and 500 mg of iron) for subjects ≥ 6 years old. Because of the possibility of dose changes at increments of 125 mg/day among subjects 6–9 years old, this age group would ideally receive formulated powder for oral suspension. Although the formulated powder for oral suspension is not an ideal pharmaceutical composition, it has been considered as an appropriate option for the pediatric population. During stage 1, SFOH was administered at an age-dependent starting dose, and titrated in increments of 125 to 500 mg iron/day, based on subject age (Supplementary Table 2). Subjects randomized to CaAc received a starting dose of 0.45 mL/kg/day of an oral solution containing 667 mg of CaAc per 5 mL (Phoslyra; Lyne Laboratories Inc., Brockton, MA 02301, USA), or an equivalent dose of their previous PB (calcium-based or sevelamer) if the investigator considered this more appropriate. CaAc was titrated in increments of 0.1 to 0.2 mL/kg/day (maximum dose of 1.25 mL/kg/day up to 35 kg, and 44 mL/day if > 35 kg).

Subsequently, doses of SFOH or CaAc were titrated as required for efficacy (to achieve age-specific target serum phosphorus levels (Fig. 1), provided a subject had been receiving a stable dose for a minimum of 2 weeks) [27]. From week 4 onwards, subjects who achieved the age-specific target serum phosphorus level could enter stage 2 of the study, where they continued receiving the same dose of SFOH or CaAc.

### Analysis populations

The safety population included all subjects who received ≥ 1 dose of study medication. The full analysis set (FAS) included all subjects randomized to treatment at stage 1 who received ≥ 1 dose of randomized treatment and who had at least 1 post-baseline assessment of the efficacy endpoint (serum phosphorus level). The per protocol set (PPS) included all subjects in the FAS who had no major protocol deviations. The FAS was the primary population for the analyses of efficacy and was also used for analyses of baseline characteristics. A subset of efficacy parameters was evaluated for the PPS population.

### Study outcomes

The primary efficacy endpoint was change in serum phosphorus levels from baseline to the end of stage 1 in the SFOH group. Secondary efficacy endpoints included change in serum phosphorus from baseline to the end of stage 1 in the CaAc group, and to the end of stage 2 in the SFOH and CaAc groups. Serum phosphorus values at each visit during stage 1 and 2, and percentage of subjects with serum phosphorus levels within the age-dependent target range, or within the age-dependent normal range at each visit were also evaluated.

Primary safety endpoints were the adverse event (AE) profile and the percentage of withdrawals due to AEs. Secondary safety endpoints included serum total corrected calcium over time; percentage of subjects who developed ≥ 1 episode of sustained hypercalcemia (elevated serum calcium confirmed by repeat sample 1 week later) after start of treatment; serum iPTH levels over time; and routine clinical laboratory tests.

### Sample size calculation and statistical analysis

#### Sample size calculation

In the SFOH treatment group, assuming a mean ± standard deviation (SD) change in serum phosphorus from baseline to end of stage 1 of 1.2 ± 2.0 mg/dL, and further allowing for an estimated dropout rate of 30%, 100 randomized subjects would provide more than 90% power to detect a significant change in serum phosphorus levels from baseline to the end of stage 1. One hundred (*n* = 100) subjects were also considered sufficient to provide robust safety and dosing information for SFOH in pediatric and adolescent subjects with CKD.

#### Primary efficacy endpoint analyses

The primary endpoint was based on central laboratory data; in case of missing data, the change from baseline was to be computed using pre- and post-treatment values from the local laboratory. Change from baseline was analyzed using a pre-defined linear mixed model, with treatment, baseline serum phosphorus, age at randomization (by category), geographic region (US and non-US), and gender as fixed effects. To account for imbalance in the age group covariate, a post hoc analysis was performed using a linear mixed model that included the same fixed effects as described above, but with age at randomization as a continuous variable. Summary statistics, with the estimate of the adjusted mean change from baseline and its 95% confidence interval (95% CI), as well as the corresponding *p* value (*t* test), were provided.

Sensitivity analyses included a repeat of the main analysis based on observed data from the central laboratory only (with no imputation using local laboratory data). Homogeneity of the primary endpoint findings was investigated through analysis of change from baseline in subgroups defined by age at randomization, serum phosphorus at baseline according to the age-related normal range, and the combination of age at randomization and serum phosphorus at baseline according to the age-related normal range.

#### Secondary efficacy endpoint analyses

Secondary efficacy endpoints related to the change from baseline in serum phosphorus were analyzed using the same pre-defined linear mixed model as for the primary efficacy endpoint; in addition, a post hoc analysis was performed as described above. Overall compliance, based on doses taken relative to the number expected to be taken, according to drug dispensing/return records, was summarized using descriptive statistics.

#### Safety analyses

Treatment-emergent adverse events (TEAEs) were summarized for both treatment groups (subject incidence rates and total number of events). Medical history and adverse events were coded using the Medical Dictionary for Regulatory Activities (version 19.1). For laboratory parameters, absolute values and changes from baseline values, based on central laboratory data, were summarized separately at each time point by treatment group. The percentage of subjects who developed ≥ 1 episode of sustained hypercalcemia after the start of treatment was summarized by treatment group.

#### General statistical methods

All descriptive statistical analyses were performed using Statistical Analysis System® statistical software (version 9.4). Formal statistical hypothesis testing was performed on the primary efficacy endpoint only, at a two-sided significance level of 0.05.

## Results

### Patient disposition and analysis sets

**Fig. 2:**
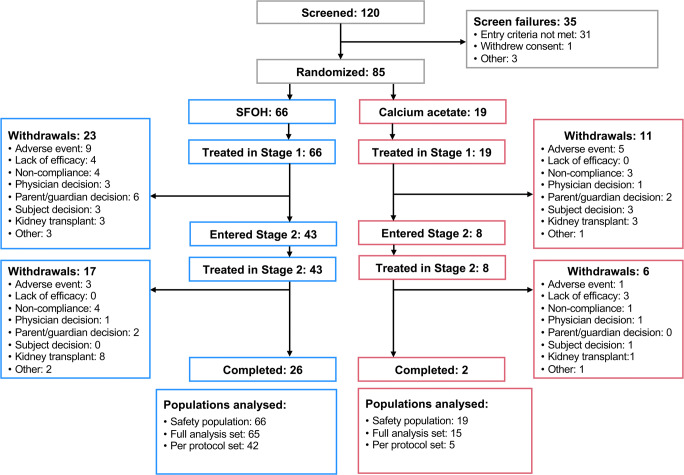
Patient disposition (stage 1 and stage 2). SFOH, sucroferric oxyhydroxide. Note: More than one reason for study withdrawal may have been given for an individual subject

Patient disposition (stage 1 and stage 2) is shown in Fig. 2. There were 85 subjects in the safety population (*n* = 66 [SFOH] and *n* = 19 [CaAc]) and 80 subjects in the FAS, which was the primary population for efficacy analysis (*n* = 65 [SFOH] and *n* = 15 [CaAc]). Major protocol deviations, mainly involving compliance with study treatment, were identified in 23 (35.4%) subjects in the SFOH group and 10 (66.7%) subjects in the CaAc group. Therefore, only 47 subjects were included in the PPS population (*n* = 42 [SFOH] and *n* = 5 [CaAc]). Baseline demographic and disease characteristics were generally comparable between treatment groups in the FAS (Table 1). Ages of subjects in the FAS ranged from 2 to ≤ 18 years and the majority (67.5%) were receiving HD. In both treatment groups, there was an imbalance in patient age group categories. Adolescents (12–≤ 18 years) comprised the majority of subjects treated with SFOH or CaAc (64.6% and 66.7%, respectively), whereas subjects aged 6–< 12 years (26.2% and 26.7%) or 2–< 6 years (9.2% and 6.7%) accounted for smaller proportions.

**Table 1:** Baseline demographics and characteristics (FAS; *N* = 80)

Parameter	SFOH (*N* = 65)	CaAc (*N* = 15)
Mean ± SD age at randomization, years	12.1 ± 4.10	12.3 ± 3.96
2–< 6 years, *n* (%)	6 (9.2)	1 (6.7)
6–< 12 years, *n* (%)	17 (26.2)	4 (26.7)
12–≤ 18 years, *n* (%)	42 (64.6)	10 (66.7)
Male, *n* (%)	31 (47.7)	5 (33.3)
Mean ± SD body weight, kg	42.1 ± 18.1	37.4 ± 19.2
Etiology of CKD, *n* (%)		
Congenital anomalies of the kidney and urinary tract	19 (29.2)	3 (20.0)
Glomerulonephritis	10 (15.4)	4 (26.7)
Hypodysplasia and reflux	2 (3.1)	2 (13.3)
Obstructive uropathy	8 (12.3)	1 (6.7)
Polycystic kidney disease	3 (4.6)	0
Other	23 (35.4)	5 (33.3)
CKD stage, *n* (%)		
4	13 (20.0)	1 (6.7)
5	52 (80.0)	14 (93.3)
Dialysis modality, *n* (%)		
Hemodialysis	45 (69.2)	9 (60.0)
Peritoneal dialysis	5 (7.7)	5 (33.3)
None	15 (23.1)	1 (6.7)
Phosphate binder-naïve, *n* (%)		
Yes	18 (27.7)	3 (20.0)
No	47 (72.3)	12 (80.0)
Washout period needed, *n* (%)		
*n* (missing) Yes	47 (18) 7 (14.9)	12 (3) 4 (33.3)
No	40 (85.1)	8 (66.7)
Mean (SD) baseline serum phosphorus, mg/dL^a^	6.41 (1.62)	6.67 (2.07)

*CaAc*, calcium acetate; *CKD*, chronic kidney disease; *FAS*, full analysis set; *SD*, standard deviation; *SFOH*, sucroferric oxyhydroxide

^a^Serum phosphorus data are from central laboratory

### Primary efficacy endpoint results

In the overall FAS, the reduction in serum phosphorus from baseline to the end of stage 1 in the SFOH group, analyzed with the pre-defined statistical model, was not statistically significant (*p* = 0.146): least squares (LS) mean ± standard error (SE) of − 0.371 ± 0.251 mg/dL (95% CI 0.874, 0.132) (Table 2). Similar results were observed from the sensitivity analysis without imputation of missing central laboratory values (data not shown), and analysis of the PPS population (Table 2). However, a post hoc analysis, accounting for imbalance in the age group covariate, showed a statistically significant reduction in serum phosphorus from baseline to the end of stage 1 in the overall FAS: LS mean ± SE of − 0.488 ± 0.186 mg/dL (95% CI − 0.861, − 0.116; *p* = 0.011), as well as in the PPS: LS mean ± SE of − 0.552 ± 0.270 mg/dL (95% CI − 1.098, − 0.005; *p* = 0.048) (Table 2).

**Table 2:** Change in serum phosphorus from baseline to end of stage 1 in the sucroferric oxyhydroxide group (FAS and PPS populations)

Parameter	FAS population (*n* = 65)	PPS population (*n* = 42)
Baseline		
Mean ± SD, mg/dL	6.45 ± 1.698	6.31 ± 1.605
End of stage 1		
Mean ± SD, mg/dL	5.90 ± 1.990	5.68 ± 2.106
Change from BL to end of stage 1		
Mean ± SD	− 0.54 ± 1.508	− 0.63 ± 1.675
Pre-defined model^a^		
LS mean ± SE	− 0.371 ± 0.251	− 0.433 ± 0.388
95% CI	− 0.874, 0.132	− 1.220, 0.353
*p* value^b^	0.146	0.271
Post hoc model^c^		
LS mean ± SE 95% CI *p* value^b^	− 0.488 ± 0.186 − 0.861, − 0.116 0.011	− 0.552 ± 0.270 − 1.098, − 0.005 0.048

*BL*, baseline; *CI*, confidence interval; *FAS*, full analysis set; *LS*, least squares; *PPS*, per protocol set; *SD*, standard deviation; *SE*, standard error

^a^Results are obtained from a linear mixed model that includes change in serum phosphorus levels from baseline to the end of stage 1 as dependent variable and treatment, baseline serum phosphorus, age (in categories) at randomization, region (non-US/US), and gender as fixed effects (pre-defined statistical model)

^b^*p* value for least squares means *t* test is presented

^c^Results are obtained from a linear mixed model which includes change in serum phosphorus levels from baseline to the end of stage 1 as dependent variable and treatment, baseline serum phosphorus, age at randomization, region (non-US/US), and gender as fixed effects (post hoc analysis)

Analyses by age group showed reductions in serum phosphorus from baseline in all age group categories (Table 3); however, statistically significant reductions (*p* = 0.022) in serum phosphorus were achieved only in subjects aged ≥ 12 to ≤ 18 years. This finding was further confirmed by the post hoc analysis.

**Table 3:** Change in serum phosphorus level from baseline to end of stage 1 in the sucroferric oxyhydroxide group by age group (FAS population; *N* = 65)

Parameter	Age ≥ 2 to < 6 years (*n* = 6)	Age ≥ 6 to < 12 years (*n* = 17)	Age ≥ 12 to ≤ 18 years (*n* = 42)
Baseline			
Mean ± SD, mg/dL	7.33 ± 1.992	6.93 ± 1.883	6.12 ± 1.522
End of stage 1			
Mean ± SD, mg/dL	7.09 ± 1.924	6.21 ± 2.730	5.61 ± 1.578
Change from BL to stage 1			
Mean ± SD	− 0.24 ± 0.783	− 0.72 ± 2.136	− 0.51 ± 1.291
Pre-defined model^a^			
LS mean ± SE	− 0.243 ± 0.380	− 0.620 ± 0.489	− 0.460 ± 0.192
95% CI	− 1.450, 0.965	− 1.675, 0.436	− 0.850, − 0.070
*p* value^b^	0.568	0.227	0.022
Post hoc model^c^			
LS mean ± SE 95% CI *p* value^b^	− 0.243 ± 0.405 − 1.986, 1.501 0.610	− 0.608 ± 0.498 − 1.694, 0.478 0.246	− 0.460 ± 0.195 − 0.856, − 0.065 0.024

*BL*, baseline; *CI*, confidence interval; *FAS*, full analysis set; *LS*, least squares; *SD*, standard deviation; *SE*, standard error

^a^Results are obtained from a linear mixed model that includes change in serum phosphorus levels from baseline to the end of stage 1 as dependent variable and treatment, baseline serum phosphorus, age (in categories) at randomization, region (non-US/US), and gender as fixed effects (pre-defined statistical model)

^b^*p* value for least squares means *t* test is presented

^c^Results are obtained from a linear mixed model which includes change in serum phosphorus levels from baseline to the end of stage 1 as dependent variable and treatment, baseline serum phosphorus, age at randomization, region (non-US/US), and gender as fixed effects (post hoc analysis)

Among subjects in the SFOH group with serum phosphorus levels above age-related normal ranges at baseline (*n* = 40), there were statistically significant reductions (*p* = 0.006) in serum phosphorus from baseline to the end of stage 1 (LS mean ± SE − 0.872 ± 0.296 mg/dL [95% CI − 1.474, − 0.270]), whereas no significant change was observed for subjects whose baseline phosphorus was within or below normal ranges (*n* = 25; LS mean ± SE 0.255 ± 0.452 mg/dL [95% CI − 0.692, 1.201]). These results were confirmed by the post hoc analysis with LS mean ± SE serum phosphorus reductions of − 0.942 ± 0.246 mg/dL (95% CI − 1.440, − 0.443; *p* = 0.0005) in SFOH subjects with serum phosphorus levels above age-related normal ranges at baseline, and no significant change in the SFOH subjects with baseline phosphorus within or below normal ranges: LS mean ± SE of 0.208 ± 0.232 mg/dL (95% CI − 0.277, 0.692).

Greater reductions from baseline in serum phosphorus were observed in all age groups of subjects with higher baseline serum phosphorus levels. However, these were only statistically significant (*p* = 0.001) among subjects aged ≥ 12 to ≤ 18 years old (Supplementary Table 3).

### Secondary efficacy endpoints

In the FAS, LS mean ± SE change in serum phosphorus from baseline to the end of stage 1 for the CaAc group (*n* = 15) was − 1.903 ± 0.992 mg/dL (95% CI − 4.147, 0.340; *p* = 0.0872) based on the pre-defined statistical model and − 0.839 ± 0.709 mg/dL (95% CI − 2.418, 0.741; *p* = 0.264) based on the post hoc analysis model. LS mean ± SE change in serum phosphorus from baseline to the end of stage 2 was + 0.307 ± 0.614 mg/dL (95% CI − 0.947, 1.560) in the SFOH group (*n* = 36) and − 1.218 ± 0.676 mg/dL (95% CI −9.804, 7.369) in the CaAc group (*n* = 6).

**Fig. 3:**
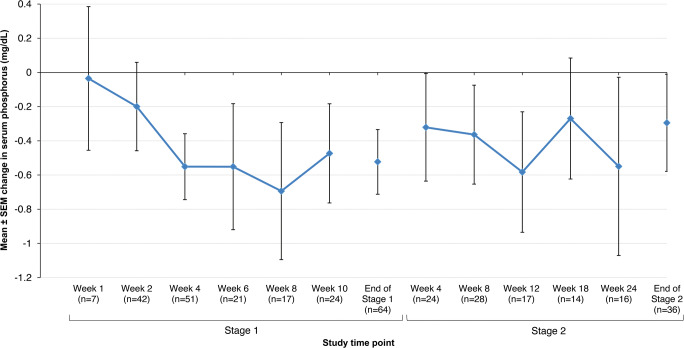
Mean change (± SEM) from baseline in serum phosphorus levels to end of stage 2 in the sucroferric oxyhydroxide group (FAS; *N* = 65). FAS, full analysis set; SEM, standard error of the mean. Data are from central laboratory

Serum phosphorus levels displayed small but consistent decreases from baseline in the SFOH group throughout the study (Fig. 3), whereas fluctuations relative to baseline were observed with CaAc, although the patient numbers in this group were small (Supplementary Fig. 2). In the FAS, the percentage of subjects in the SFOH group with serum phosphorus levels within age-related target ranges increased from 16.9% at baseline to 39.1% by the end of stage 1, and to 35.0% by the end of stage 2 (Supplementary Fig. 3). Similar increases were also observed in the PPS population: 21.4% at baseline, 45.2% by the end of stage 1, and 39.3% by the end of stage 2.

### Treatment compliance

Mean ± SD treatment compliance during the overall study period (stage 1 and stage 2) in the safety population was 79.9 ± 23.7% in the SFOH group and 62.6 ± 37.9% in the CaAc group. Analyses by age group showed that median compliance with SFOH treatment during the overall study period ranged from 77.9% (≥ 12 to ≤ 18 years subgroup) to 93.3% (≥ 2 to < 6 years subgroup) (Supplementary Table 4).

### Safety

The mean ± SD duration of treatment exposure was longer in the SFOH group than in the CaAc group, both during stage 1 (45.7 ± 23.2 days vs. 37.8 ± 28.4 days, respectively) and the overall study (126.5 ± 83.9 days vs. 73.9 ± 73.6 days). Mean ± SD average daily dose in the SFOH group was 1216.2 ± 526.4 mg iron during stage 1 and 1281.4 ± 611.8 mg iron for the overall study; in the CaAc group, mean average daily dose ± SD was 12.4 ± 8.1 mL during stage 1 and 12.3 ± 8.8 mL for the overall study. The mean ± SD prescribed average daily dose of SFOH during the overall study increased with age, ranging from 636.5 ± 260.1 mg iron, in subjects aged ≥ 2 to < 6 years, to 1798.9 ± 483.9 mg iron in subjects aged ≥ 12 to ≤ 18 years (Supplementary Table 4). The mean average daily number of tablets/powders taken by subjects was low and comparable across all age subgroups, ranging from 2.95 to 3.47 tablets/sachets per day. The mean ± SD prescribed daily dose of CaAc also increased with age, although the number of patients in each age subgroup was small (Supplementary Table 5).

The proportion of subjects experiencing at least one TEAE was comparable between the groups for the overall study period (SFOH 75.8% and CaAc 73.7%) (Table 4). A summary of TEAEs occurring in ≥ 5% patients in either treatment group during the overall study is provided in Supplementary Table 6. The proportion of subjects experiencing at least one treatment-related TEAE during the overall study was comparable between treatment groups (SFOH 39.4% subjects; CaAc 36.8% subjects). The most common treatment-related TEAEs occurred in the system organ class of GI disorders and metabolism and nutrition disorders (Supplementary Table 7). GI disorders considered related to study treatment were reported in 22 (33.3%) subjects in the SFOH group and 4 (21.1%) subjects in the CaAc group. The incidence of treatment-related TEAEs of diarrhea, discolored feces, and gastritis was higher in the SFOH group, while the incidence of nausea, abdominal pain, constipation, and upper abdominal pain was higher in the CaAc group. No TEAEs leading to death were reported in either treatment group. The percentage of subjects with serious TEAEs was higher for SFOH (27.3%) than for CaAc (15.8%) during the overall study. Five serious TEAEs in 3 subjects, all in the SFOH group, were considered by the investigators as possibly related to study treatment: ileus (1 subject); gastritis (1 subject); and blood pressure increased, hypertension, and vena cava thrombosis (1 subject).

**Table 4:** Overview of treatment-emergent adverse events in either treatment group during the study (safety population; *n* = 85)

	SFOH (*N* = 66)		CaAc (*N* = 19)	
	Patients, *n* (%)	Events, *n*	Patients, *n* (%)	Events, *n*
End of stage 1				
Any TEAE	42 (63.6)	123	13 (68.4)	46
Any treatment-related TEAE	24 (36.4)	43	7 (36.8)	13
Any serious TEAE	13 (19.7)	19	3 (15.8)	7
Any TEAE leading to death	0	0	0	0
Any TEAE leading to study drug withdrawal	11 (16.7)	17	6 (31.6)	8
End of stage 2				
Any TEAE	50 (75.8)	204	14 (73.7)	63
Any treatment-related TEAE	26 (39.4)	50	7 (36.8)	13
Any serious TEAE	18 (27.3)	43	3 (15.8)	9
Any TEAE leading to death	0	0	0	0
Any TEAE leading to study drug withdrawal	12 (18.2)	19	6 (31.6)	8

*CaAC*, calcium acetate; *SFOH*, sucroferric oxyhydroxide; *TEAE*, treatment-emergent adverse event

The proportion of patients who withdrew due to TEAEs was higher in the CaAc group (*n* = 6, 31.6%) than in the SFOH group (*n* = 12, 18.2%). Events that led to drug withdrawal in more than one subject in the SFOH group were diarrhea (6 subjects), nausea (2 subjects), and vomiting (2 subjects); those in the CaAc group were hypercalcemia (3 subjects) and hyperphosphatemia (2 subjects).

Changes in serum total corrected calcium, calcium-phosphorus product, iPTH ferritin, and iron are shown in Supplementary Table 8. Changes from baseline in serum total corrected calcium were minimal in both treatment groups. The incidence of sustained hypercalcemia was higher in the CaAc group than in the SFOH group (21.1% vs. 9.1% subjects experienced ≥ 1 episode of sustained hypercalcemia during the study). Calcium-phosphorus product was lower and decreased from baseline to a greater extent in the SFOH group than in the CaAc group. No clinically meaningful changes from baseline in serum iPTH were observed in either group during the study. Observed mean changes from baseline in ferritin were similar between subjects in the SFOH and CaAc groups, with the caveat that approximately half of the patients were receiving oral or intravenous iron. Although some changes from baseline and individual deviations from the normal ranges were observed for other clinical laboratory parameters, including liver enzymes, vitamin levels, and bone markers (alkaline phosphatase and bone-specific alkaline phosphatase), they showed no trends or clinically relevant changes (data available on ClinicalTrials.gov).

## Discussion

The aim of this phase 3 clinical trial was to investigate the safety and efficacy of SFOH in pediatric and adolescent patients with CKD, and to provide dosing information for this patient population.

A calcium-based PB (CaAc) was chosen as an active control to provide comparisons of the serum phosphorus-lowering effects and adverse event profile of SFOH with those of a PB commonly used in clinical practice. No formal comparison with CaAc was planned. The study planned to randomize approximately 130 subjects, but was prematurely ended due to challenges with patient recruitment, especially in patients below 9 years of age, and modification of study requirements, as agreed with the US Food and Drug Administration (FDA) and the European Medicines Agency (EMA).

The adverse event profile of SFOH in pediatric and adolescent subjects with CKD was generally consistent with the known safety profile of SFOH and with previous experience in adults with kidney failure [21–23]. Most reported adverse events in the present study were characteristic of this patient population (i.e., related to dialysis or underlying concomitant diseases), or were consistent with the safety profile of SFOH in adults. No new safety signals were identified, compared with the adult population, and no clear safety difference was observed between age groups; however, the number of subjects in the younger age groups was small. Diarrhea was reported as a treatment-related TEAE in 16.7% of subjects randomized to SFOH. This observation is consistent with phase 2 and phase 3 clinical trials of SFOH in adults [21–23], although the reporting rate of 11.6% in the pooled adult studies [28] was slightly lower than that seen in this pediatric study. The majority of diarrhea events were graded as mild or moderate in intensity, which is consistent with the typical safety profile for adults. Other than diarrhea, no other treatment-related TEAE was reported as “very common” (≥ 10% frequency). The percentage of withdrawals due to TEAEs was lower in the SFOH group than in the CaAc group.

The analysis of efficacy in the overall SFOH group (primary endpoint) using the pre-defined statistical model did not show statistical significance; this model was not the most appropriate given the dichotomization of subject age and imbalanced age categories, which reduced the observed treatment effect size. Statistically significant reductions in serum phosphorus were observed in a post hoc analysis using a statistical model that took into account the imbalance of age categories; the post hoc analysis findings provide an accurate reflection of the treatment effect with SFOH in this study population.

Overall, reductions in serum phosphorus levels were observed with SFOH in all age groups evaluated; however, a statistically significant reduction was observed only in the subgroup of subjects aged ≥ 12 to ≤ 18 years. A clinically important and statistically significant decrease in serum phosphorus from baseline to the end of stage 1 was observed in the subgroup of SFOH subjects who had serum phosphorus above the age-related normal range at baseline, but not in the subgroup with serum phosphorus within or below the age-related normal range at baseline. The last group accounted for 25/65 (38.5%) subjects in the SFOH group, which may have reduced the magnitude of the serum phosphorus-lowering effect observed in the overall SFOH group. Subgroup analysis by both age group and baseline serum phosphorus level showed statistically significant reductions in serum phosphorus levels among subjects aged ≥ 12 to ≤ 18 years old with baseline phosphorus above age-related normal ranges, but not among subjects in the same age group with baseline levels below or within normal ranges. The statistically significant reductions in serum phosphorus in subjects with serum phosphorus levels at baseline above the age-related normal range are consistent with results observed for sevelamer carbonate in pediatric patients [29]. However, the median dose of sevelamer carbonate in this study was 8 to 9 tablets per day.

Certain aspects of the study design probably contributed to the efficacy findings by reducing the observed treatment effect. There was overlap between the target serum phosphorus levels required for randomization per protocol (during the washout period) and the post-randomization target range. Consequently, it was possible for subjects to have had serum phosphorus levels within the post-randomization target ranges after little or no change from their baseline levels. Furthermore, the study protocol allowed a direct switch of PB without a washout period for subjects who had serum phosphorus levels above the threshold required for randomization (> 80% of patients in the study received a prior PB treatment). Therefore, for most patients who were not PB-naïve, the efficacy of SFOH was being compared to the patient’s pre-study binder therapy.

Treatment compliance was probably an additional contributor to the effect of SFOH on serum phosphorus. Non-adherence to treatment is an important determinant of PB treatment efficacy [30, 31], and was a common protocol deviation in the SFOH treatment group, identified for 27.7% of subjects in the FAS.

The efficacy of SFOH in reducing serum phosphorus levels was previously confirmed in global clinical studies in adult CKD patients on dialysis [21, 22]. The mechanism of action of SFOH is based on the binding of phosphate in the GI tract, and there is no reason to expect differences between adults and children with respect to phosphate binding. The present study confirms that SFOH works in the pediatric population. A significant treatment effect was observed in adolescents (≥ 12 to ≤ 18 years); in the younger age groups, the numeric trends were similar, but the small patient numbers made it challenging to demonstrate a statistically significant decrease of serum phosphorus in the younger patients.

Despite the limitations of this study, the results demonstrate the overall efficacy of SFOH for reducing serum phosphorus in the pediatric population and maintaining age-appropriate serum phosphorus levels; the proportion of subjects achieving serum phosphorus levels within the age-related target range increased more than twofold from baseline to the end of stage 1 and stage 2.

Studies of adults with CKD have demonstrated that SFOH is effective for lowering serum phosphorus and achieves this with a low daily pill burden. This low pill burden was confirmed in the current pediatric study (Supplementary Table 4), and may increase long-term adherence compared to other PBs.

## Conclusions

Overall, the safety profile of SFOH in pediatric and adolescent subjects with CKD was comparable to that previously observed in adult subjects with kidney failure. No new safety signals were identified in the pediatric and adolescent populations. Serum phosphorus-lowering trends were observed in all age groups. Statistically significant reductions in serum phosphorus with SFOH were observed in adolescent subjects aged ≥ 12 to ≤ 18 years old, and in the group of subjects with baseline serum phosphorus levels above age-related normal ranges. SFOH demonstrated a positive benefit–risk profile in the pediatric population with a low pill burden, and the results of this study support for the use of SFOH in pediatric and adolescent CKD patients with hyperphosphatemia.

## Electronic supplementary material

ESM 1 (DOCX 464 kb).
