# Supplementary material for: Safety and efficacy of sucroferric oxyhydroxide in pediatric patients with chronic kidney disease
Source: Pediatr Nephrol. 2020 Oct 27;36(5):1233–44. doi: 10.1007/s00467-020-04805-y (PMC8009783; doi:10.1007/s00467-020-04805-y)
Supplement: Supplementary file 1 — (DOCX 464 kb). [file 467_2020_4805_MOESM1_ESM.docx]

**Supplementary materials**

**Supplementary Figure 1**

a. Schedule of events


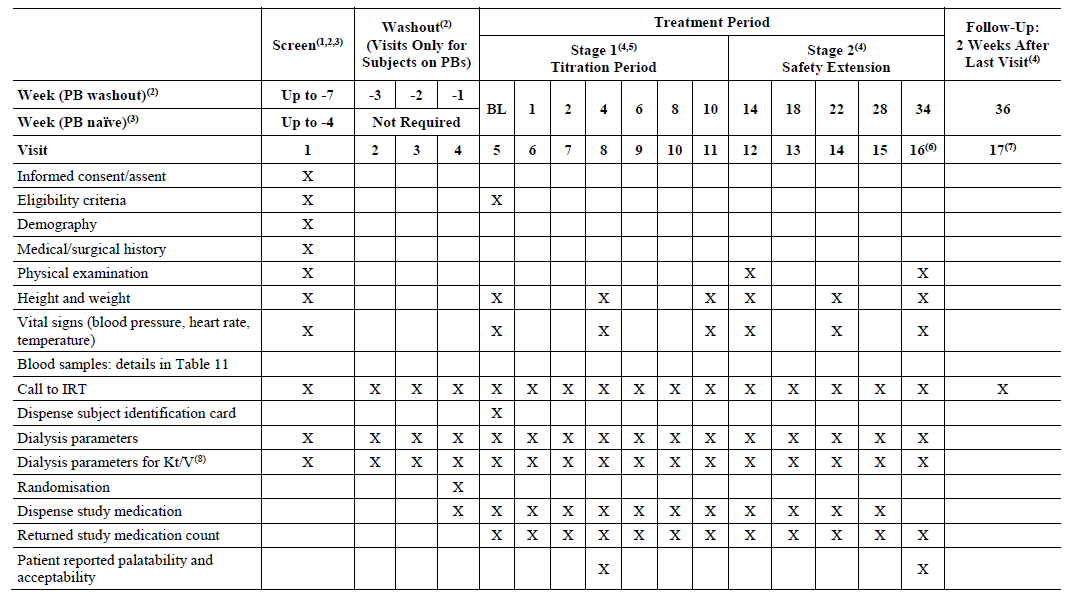


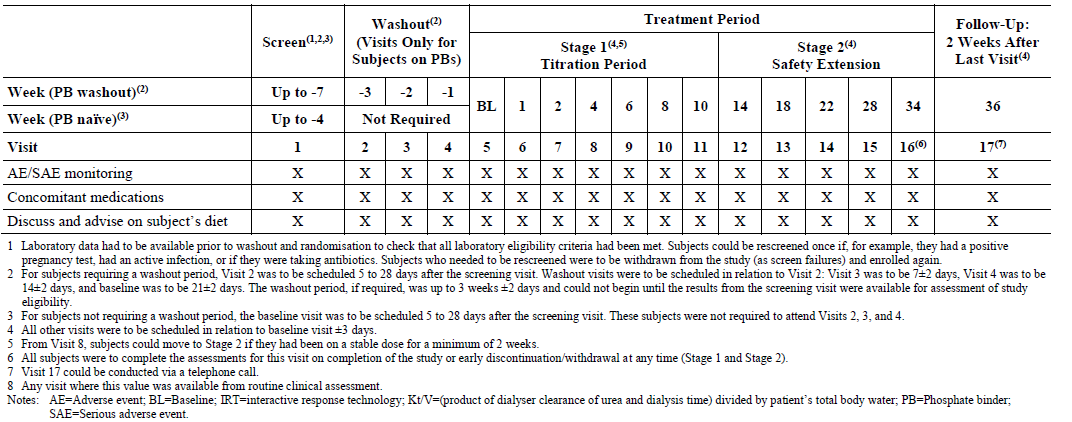


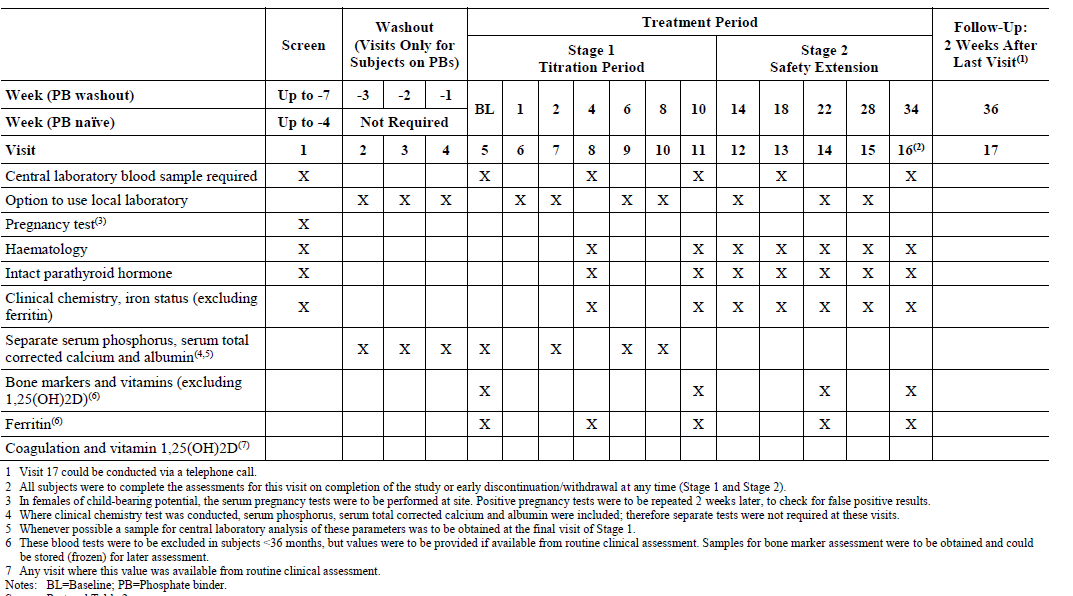
**b. Summary of blood samples**

**Supplementary Figure 2.** Mean change (± SEM) from baseline in serum phosphorus levels to end of Stage 2 in the CaAc group (FAS; n=15)


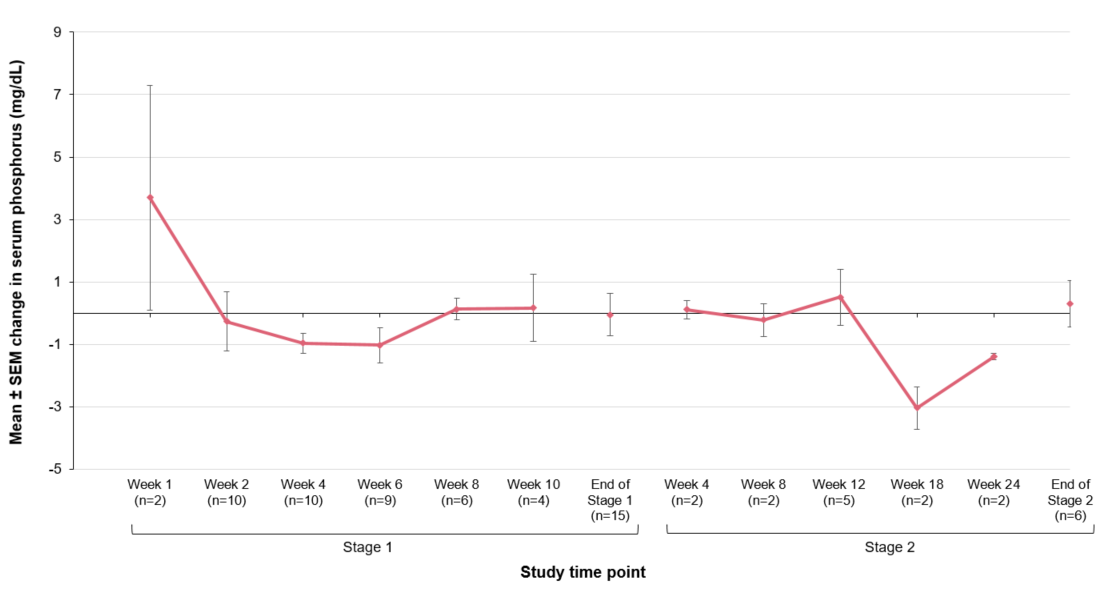


Data are from central laboratory.

CaAc, calcium acetate; FAS, full analysis set; SEM, standard error of the mean

**Supplementary Figure 3:** Percentage of subjects in the sucroferric oxyhydroxide group with serum phosphorus level within age-related target range over time (FAS; N=65)


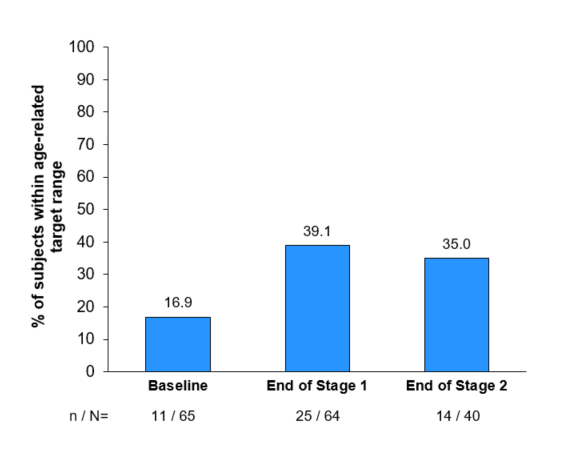


FAS, full analysis set

**Tables**

**Supplementary Table 1: Study exclusion criteria**

| **Subjects were excluded from participation if any of the following exclusion criteria were met:** |
| --- |
| 1. Subjects with hypercalcemia at screening  2. Subjects with intact parathyroid hormone (iPTH) levels >700 pg/mL at screening.  3. Subjects who were PB-naïve who weighed <5 kg at screening, or subjects receiving stable doses of PBs who weighed <6 kg at screening (in order to comply with maximum blood sample volumes in pediatric clinical trials).  4. Subjects requiring feeding tube sizes ≤6 FR (French catheter scale).  5. Subjects with planned or expected parathyroidectomy within the next 12 months, in the Investigator’s opinion.  6. Subjects with history of:   - Major GI surgery which, in the Investigator’s opinion, was likely to influence the outcome of treatment with PBs. - Significant GI disorders.   7. Subjects with estimated life expectancy of less than 12 months.  8. Subjects with known seropositivity to human immunodeficiency virus.  9. Subjects with a history of hemochromatosis or other iron accumulation disorders.  10. Subjects on PD with a history of peritonitis in the last 3 months or ≥3 episodes in the last 12 months.  11. Subjects with hypocalcemia (serum total corrected calcium <1.9 mmol/l; <7.6 mg/dL) at screening.  12. Subjects with raised alanine aminotransferase or aspartate aminotransferase >3 times the upper limit of the normal range based on central laboratory results at screening.  13. Subjects taking more than 2 PBs concomitantly prior to screening.  14. Subjects taking any prohibited medication(s)  15. Subjects with known hypersensitivity and/or intolerance to any of the active substances or to any of the excipients to be administered.  16. Subjects previously randomized into this study.  17. Subjects enrolled in or having completed any other investigational device or drug study <30 days prior to screening, or receiving other investigational agent(s).  18. Subjects who were pregnant (e.g., positive human chorionic gonadotropin test) or breastfeeding.  19. If of child-bearing potential, subjects not using adequate contraceptive precautions. Subjects had to agree to use a highly effective method of birth control during the study and for 1 month after the last dose of study medication. Adequate methods of birth control were defined as those which result in a low failure rate (i.e., <1% per year) when used consistently and correctly such as implants, injectables, combined oral contraceptives, some intra-uterine devices, sexual abstinence, or vasectomized partner. Non-child-bearing potential included being surgically sterilized at least  6 months prior to the study.  20. Subjects with a history of drug or alcohol abuse within 2 years prior to screening.  21. Subjects with significant medical condition(s) e.g., uncontrolled diabetes, known hepatitis B surface antigen positivity and/or hepatitis C virus ribonucleic acid positivity, anticipated need for major surgery during the study, or any other kind of disorder that could be associated with increased risk to the subject, or could interfere with study assessments or outcomes.  GI, gastrointestinal; PB, phosphate binder; PD, peritoneal dialysis. |

**Supplementary Table 2:** Sucroferric oxyhydroxide dosing during the study

|  | **Sucroferric oxyhydroxide total daily dose (mg iron/day)** | | |
| --- | --- | --- | --- |
| **Age** | **Starting dose** | **Dose change** | **Maximum dose** |
| 0 to <1 year | 125 | 125 or 250 | 1,000 |
| ≥1 year to <6 years | 500 | 125 or 250 | 1,250 |
| ≥6 years to <9 years | 750 | 125, 250 or 375 | 2,500 |
| ≥9 years to <18 years | 1,250 | 250 or 500 | 3,000 |

**Supplementary Table 3:** Change in serum phosphorus from baseline to end of Stage 1 in the SFOH group, by age at randomization and serum phosphorus at baseline according to age-related normal range (FAS; N=65)

|  | **≥2 to <6 Years** | | **≥6 to <12 Years** | | **≥12 to ≤18 Years** | |  |
| --- | --- | --- | --- | --- | --- | --- | --- |
| **Time point** | **Baseline phosphorus above normal range  (n=5)** | **Baseline phosphorus below or within normal range (n=1)** | **Baseline phosphorus above normal range  (n=12)** | **Baseline phosphorus below or within normal range (n=5)** | **Baseline phosphorus above normal range  (n=23)** | **Baseline phosphorus below or within normal range  (n=19)** |  |
| Baseline |  |  |  |  |  |  |  |
| Mean ± SD, mg/dL | 7.96 ± 1.428 | 4.21 | 7.69 ± 1.562 | 5.10 ± 1.240 | 7.17 ± 1.241 | 4.86 ± 0.564 |  |
| End of stage 1 |  |  |  |  |  |  |  |
| Mean ± SD, mg/dL | 7.55 ± 1.747 | 4.80 | 6.52 ± 3.140 | 5.47 ± 1.308 | 6.11 ± 1.736 | 5.01 ± 1.136 |  |
| Change from BL to Stage 1 |  |  |  |  |  |  |  |
| Mean ± SD | –0.41 ± 0.748 | 0.59 | –1.17 ± 2.388 | 0.37 ± 0.692 | –1.06 ± 1.114 | 0.15 ± 1.199 |  |
| LS mean ± SE^a^ | –0.472 ± 0.415 | 0.588 ± 0.000 | –1.169 ± 0.650 | 0.369 ± 0.221 | –0.965 ± 0.252 | 0.146 ±0.287 |  |
| 95% CI | –2.258, 1.313 | NA, NA | –2.667, 0.330 | –2.444, 3.183 | –1.492, –0.438 | –0.467, 0.758 |  |
| *P* value^b^ | 0.3730 |  | 0.110 | 0.344 | 0.001 | 0.620 |  |

BL, baseline; CI, confidence interval; FAS, full analysis set; LS, least squares; SD, standard deviation; SE, standard error;
SFOH, sucroferric oxyhydroxide; NA, Not Applicable

^a^Results are obtained from a linear mixed model that includes change in serum phosphorus levels from baseline to the end of Stage 1 as dependent variable and treatment, baseline serum phosphorus, age (in categories) at randomization, region (non-US/US) and gender as fixed effects.

^b^p value for least squares means *t* test is presented. **Supplementary Table 4:** Exposure to sucroferric oxyhydroxide by age group during the overall study (Safety population; N=66)

|  | Age group | | | |
| --- | --- | --- | --- | --- |
|  | **≥2 to <6 years** | **≥6 to 9 years** | **≥9 to 12 years** | **≥12 to ≤18 years** |
| Actual average daily number of tablets/powders^a^ during Stage 1 | (N=5) | (N=7) | (N=10) | (N=43) |
| Mean ± SD | 2.87 ± 1.017 | 3.24 ± 0.590 | 3.12 ± 0.439 | 3.23 ± 1.295 |
| Median | 3.38 | 3.01 | 3.11 | 3.18 |
| Actual average daily number of tablets/powders^a^ overall study | (N=5) | (N=7) | (N=10) | (N=43) |
| Mean ± SD | 2.95 ± 1.438 | 3.47 ± 0.936 | 3.44 ± 0.824 | 3.19 ± 1.389 |
| Median | 3.38 | 3.26 | 3.67 | 3.00 |
| Prescribed average daily  dosage (mg iron) during Stage 1 | (N=6) | (N=7) | (N=10) | (N=43) |
| Mean ± SD | 573.67 ± 118.638 | 867.33 ± 133.329 | 1362.29 ± 288.414 | 1600.93 ± 340.306 |
| Median | 500.00 | 895.83 | 1293.18 | 1535.21 |
| Prescribed average daily  dosage (mg iron) overall study | (N=6) | (N=7) | (N=10) | (N=43) |
| Mean ± SD | 636.48 ± 260.137 | 1084.83 ± 354.347 | 1586.78 ± 514.354 | 1798.86 ± 483.908 |
| Median | 500.00 | 1007.00 | 1647.32 | 1760.42 |
| Compliance^b^ for Stage 1 | (N=5) | (N=7) | (N=10) | (N=43) |
| Median | 93.31% | 97.3% | 88.4% | 90.2% |
| Overall Compliance^b^ | (N=5) | (N=7) | (N=10) | (N=43) |
| Median | 93.31% | 89.4% | 87.4% | 77.9% |

SD, standard deviation

a. Actual exposure data and compliance data are classified as missing in cases where all dispensed drug was reported as lost and without a returned date collected. Note that the age groups ≥6 to 9 years and ≥9 to 12 years (instead of ≥6 to 12 years) were included as a *post hoc* analysis for more granularity of exposure data in children below the age of 12 years and for consistency with the dosing in the study, which differed for children aged below and above 9 years (see Supplementary Table 2).

b. Compliance is defined as 100 x the total number of mg of iron received (calculated from the number of tablets/sachets dispensed – unused sachets/tablets returned – unused sachets/tablets reported as lost) divided by the total prescribed dose in mg iron.

**Supplementary Table 5:** Exposure to CaAc by age group during Stage 1 and overall study (Safety population)

|  | Age group | | |
| --- | --- | --- | --- |
|  | **≥2 to <6 years** | **≥6 to 12 years** | **≥12 to ≤18 years** |
| Actual average daily dosage (mL) during Stage 1 | (N=1) | (N=5) | (N=12) |
| Mean ± SD | 4.17 | 9.99 ± 7.464 | 14.11 ± 8.271 |
| Median | 4.17 | 9.63 | 14.39 |
| Actual average daily dosage (mL) overall study | (N=1) | (N=5) | (N=12) |
| Mean ± SD | 4.17 | 10.22 ± 8.633 | 13.79 ± 9.039 |
| Median | 4.17 | 7.64 | 13.23 |
| Prescribed average daily  dosage (mL) during Stage 1 | (N=1) | (N=5) | (N=13) |
| Mean ± SD | 6.50 | 14.24 ± 6.437 | 22.44 ± 10.010 |
| Median | 6.50 | 11.00 | 24.00 |
| Prescribed average daily  dosage (mL) overall study | (N=1) | (N=5) | (N=13) |
| Mean ± SD | 6.50 | 14.07 ± 7.163 | 25.10 ± 11.722 |
| Median | 6.50 | 11.00 | 24.00 |
| Compliance^a^ for Stage 1 | (N=1) | (N=5) | (N=12) |
| Median | 64.1% | 78.1% | 50.7% |
| Overall Compliance^a^ | (N=1) | (N=5) | (N=12) |
| Median | 64.1% | 87.4% | 42.4% |

CaAc, calcium acetate; SD, standard deviation

^a^The compliance is defined as 100 x the total amount of CaAc received in mL (calculated from the number of bottles dispensed – number of unused bottles returned – number of bottles reported as lost – amount of treatment left from the partially used bottles returned) divided by the total prescribed dose in mL.
Actual exposure data and compliance are set to missing in case all dispensed drug was reported as lost and without returned date collected.

**Supplementary Table 6:** Incidence of treatment-emergent adverse events occurring in ≥5% patients in either treatment arm, by the end of Stage 2 by system organ class and preferred term (Safety population, N=85)

| **System Organ Class**  **Preferred Term** | **SFOH (N=66)** | | **CaAc (N=19)** | |
| --- | --- | --- | --- | --- |
|  | **Patients, n (%)** | **Events, n** | **Patients, n (%)** | **Events, n** |
| Any treatment-emergent adverse events | 50 (75.8) | 204 | 14 (73.7) | 63 |
| Gastrointestinal disorders | 31 (47.0) | 51 | 7 (36.8) | 10 |
| Diarrhea | 12 (18.2) | 14 | 0 (0.0) | 0 |
| Nausea | 8 (12.1) | 10 | 2 (10.5) | 2 |
| Vomiting | 6 (9.1) | 6 | 3 (15.8) | 4 |
| Constipation | 4 (6.1) | 4 | 1 (5.3) | 1 |
| Abdominal pain | 3 (4.5) | 3 | 1 (5.3) | 1 |
| Abdominal pain upper | 2 (3.0) | 2 | 1 (5.3) | 1 |
| Small intestinal perforation | 0 (0.0) | 0 | 1 (5.3) | 1 |
| Infections and infestations | 16 (24.2) | 31 | 9 (47.4) | 14 |
| Urinary tract infection | 3 (4.5) | 5 | 2 (10.5) | 3 |
| Upper respiratory tract infection | 2 (3.0) | 2 | 1 (5.3) | 2 |
| Gastroenteritis | 1 (1.5) | 1 | 1 (5.3) | 1 |
| Pharyngitis | 1 (1.5) | 1 | 1 (5.3) | 1 |
| *Clostridium difficile* colitis | 0 (0.0) | 0 | 1 (5.3) | 1 |
| *Clostridium difficile* infection | 0 (0.0) | 0 | 1 (5.3) | 1 |
| Conjunctivitis | 0 (0.0) | 0 | 1 (5.3) | 1 |
| Cystitis | 0 (0.0) | 0 | 1 (5.3) | 1 |
| Hand, foot and mouth disease | 0 (0.0) | 1 | 1 (5.3) | 1 |
| Respiratory syncytial virus infection | 0 (0.0) | 0 | 1 (5.3) | 1 |
| Staphylococcal bacteremia | 0 (0.0) | 0 | 1 (5.3) | 1 |
| Metabolism and nutrition disorders | 15 (22.7) | 23 | 9 (47.4) | 13 |
| Hypercalcemia | 4 (6.1) | 4 | 4 (21.1) | 4 |
| Hyperphosphatemia | 3 (4.5) | 3 | 3 (15.8) | 3 |
| Hyperkalemia | 2 (3.0) | 3 | 1 (5.3) | 1 |
| Dehydration | 1 (1.5) | 1 | 1 (5.3) | 1 |
| Electrolyte imbalance | 0 (0.0) | 0 | 1 (5.3) | 1 |
| Hypophosphatemia | 0 (0.0) | 0 | 1 (5.3) | 1 |
| Iron deficiency | 0 (0.0) | 0 | 1 (5.3) | 1 |
| Metabolic acidosis | 0 (0.0) | 0 | 1 (5.3) | 1 |
| General disorders and administration site conditions | 10 (15.2) | 16 | 3 (15.8) | 3 |
| Pyrexia | 3 (4.5) | 3 | 2 (10.5) | 2 |
| Catheter site hemorrhage | 0 (0.0) | 0 | 1 (5.3) | 1 |
| Vascular disorders | 9 (13.6) | 16 | 1 (5.3) | 1 |
| Hypertension | 6 (9.1) | 9 | 0 (0.0) | 0 |
| Malignant hypertension | 0 (0.0) | 0 | 1 (5.3) | 1 |
| Investigations | 8 (12.1) | 13 | 3 (15.8) | 4 |
| Weight decreased | 1 (1.5) | 1 | 1 (5.3) | 1 |
| Blood lactate dehydrogenase increased | 0 (0.0) | 0 | 1 (5.3) | 1 |
| Blood phosphorus increased | 0 (0.0) | 0 | 1 (5.3) | 1 |
| Liver function test increased | 0 (0.0) | 0 | 1 (5.3) | 1 |
| Renal and urinary disorders | 6 (9.1) | 10 | 2 (10.5) | 2 |
| Dysuria | 1 (1.5) | 1 | 1 (5.3) | 1 |
| Hematuria | 0 (0.0) | 0 | 1 (5.3) | 1 |
| Blood and lymphatic system disorders | 5 (7.6) | 6 | 0 (0.0) | 0 |
| Injury, poisoning and procedural complications | 5 (7.6) | 6 | 0 (0.0) | 0 |
| Respiratory, thoracic and mediastinal disorders | 5 (7.6) | 5 | 2 (10.5) | 4 |
| Cough | 1 (1.5) | 1 | 2 (10.5) | 2 |
| Sinus congestion | 0 (0.0) | 0 | 1 (5.3) | 2 |
| Nervous system disorders | 4 (6.1) | 5 | 2 (10.5) | 2 |
| Headache | 2 (3.0) | 2 | 1 (5.3) | 1 |
| Dizziness | 0 (0.0) | 0 | 1 (5.3) | 1 |
| Endocrine disorders | 3 (4.5) | 5 | 2 (10.5) | 2 |
| Hyperparathyroidism | 2 (3.0) | 3 | 1 (5.3) | 1 |
| Hyperparathyroidism secondary | 1 (1.5) | 2 | 1 (5.3) | 1 |
| Product issues | 3 (4.5) | 4 | 1 (5.3) | 2 |
| Device occlusion | 0 (0.0) | 0 | 1 (5.3) | 2 |
| Skin and subcutaneous tissue disorders | 3 (4.5) | 3 | 3 (15.8) | 4 |
| Excessive granulation tissue | 0 (0.0) | 0 | 1 (5.3) | 1 |
| Pruritus | 0 (0.0) | 0 | 1 (5.3) | 1 |
| Rash | 0 (0.0) | 0 | 1 (5.3) | 2 |
| Ear and labyrinth disorders | 1 (1.5) | 1 | 1 (5.3) | 1 |
| Otorrhea | 0 (0.0) | 0 | 1 (5.3) | 1 |
| Reproductive system and breast disorders | 0 (0.0) | 0 | 1 (5.3) | 1 |
| Amenorrhea | 0 (0.0) | 0 | 1 (5.3) | 1 |

CaAc, calcium acetate; SFOH, sucroferric oxyhydroxide; TEAE, treatment-emergent adverse event

N=total number of subjects; n=number of subjects

**Supplementary Table 7:** Treatment-related TEAEs until the end of Stage 2 by system organ class and preferred term (Safety population; N=85)

| **System Organ Class**  **Preferred Term** | **SFOH (N=66)** | | **CaAc (N=19)** | |
| --- | --- | --- | --- | --- |
|  | **Patients, n (%)** | **Events, n** | **Patients, n (%)** | **Events, n** |
| Any treatment-related TEAEs | 26 (39.4) | 50 | 7 (36.8) | 13 |
| Gastrointestinal disorders | 22 (33.3) | 35 | 4 (21.1) | 6 |
| Diarrhea | 11 (16.7) | 13 | 0 (0.0) | 0 |
| Nausea | 4 (6.1) | 4 | 2 (10.5) | 2 |
| Vomiting | 4 (6.1) | 4 | 1 (5.3) | 1 |
| Abdominal pain | 3 (4.5) | 3 | 1 (5.3) | 1 |
| Constipation | 3 (4.5) | 3 | 1 (5.3) | 1 |
| Feces discolored | 2 (3.0) | 2 | 0 (0.0) | 0 |
| Gastritis | 2 (3.0) | 2 | 0 (0.0) | 0 |
| Abdominal pain upper | 1 (1.5) | 1 | 1 (5.3) | 1 |
| Feces soft | 1 (1.5) | 1 | 0 (0.0) | 0 |
| Ileus | 1 (1.5) | 1 | 0 (0.0) | 0 |
| Lip swelling | 1 (1.5) | 1 | 0 (0.0) | 0 |
| Metabolism and nutrition disorders | 5 (7.6) | 5 | 5 (26.3) | 5 |
| Hyperphosphatemia | 2 (3.0) | 2 | 1 (5.3) | 1 |
| Decreased appetite | 1 (1.5) | 1 | 0 (0.0) | 0 |
| Dehydration | 1 (1.5) | 1 | 0 (0.0) | 0 |
| Hypercalcemia | 1 (1.5) | 1 | 4 (21.1) | 4 |
| Investigations | 3 (4.5) | 4 | 1 (5.3) | 1 |
| Blood creatine phosphokinase increased | 1 (1.5) | 1 | 0 (0.0) | 0 |
| Blood phosphorus decreased | 1 (1.5) | 1 | 0 (0.0) | 0 |
| Blood pressure increased | 1 (1.5) | 1 | 0 (0.0) | 0 |
| Weight decreased | 1 (1.5) | 1 | 0 (0.0) | 0 |
| Blood phosphorus increased | 0 (0.0) | 0 | 1 (5.3) | 1 |
| Endocrine disorders | 1 (1.5) | 2 | 0 (0.0) | 0 |
| Hyperparathyroidism | 1 (1.5) | 2 | 0 (0.0) | 0 |
| Respiratory, thoracic and mediastinal disorders | 1 (1.5) | 1 | 0 (0.0) | 0 |
| Hyperactive pharyngeal reflex | 1 (1.5) | 1 | 0 (0.0) | 0 |
| Vascular disorders | 1 (1.5) | 3 | 0 (0.0) | 0 |
| Hypertension | 1 (1.5) | 2 | 0 (0.0) | 0 |
| Vena cava thrombosis | 1 (1.5) | 1 | 0 (0.0) | 0 |
| Skin and subcutaneous tissue disorders | 0 (0.0) | 0 | 1 (5.3) | 1 |
| Rash | 0 (0.0) | 0 | 1 (5.3) | 1 |

CaAc, calcium acetate; SFOH, sucroferric oxyhydroxide; TEAE, treatment-emergent adverse event

N=total number of subjects; n=number of subjects

The treatment-related TEAEs were determined by the Investigator and defined as certainly, probably/likely, or possibly related to the study drug.

**Supplementary Table 8:** Mean ± SD observed values of serum total corrected calcium, calcium-phosphorus product, iPTH, ferritin and iron at the end of Stage 1 and Stage 2, and changes from baseline (Safety population; N=85)

|  | **SFOH (N=66)** | | **CaAc (N=19)** | |
| --- | --- | --- | --- | --- |
| **Parameter**  **Mean ± SD** | **Observed value** | **Change from  baseline** | **Observed value** | **Change from  baseline** |
| **Total corrected calcium, mg/dL** |  |  |  |  |
| Baseline | n=66 9.53 ± 0.575 | — | n=19  9.51 ± 0.742 | — |
| End of Stage 1 | n=65  9.41 ± 0.850 | n=65  –0.12 ± 0.897 | n=17  9.26 ± 0.523 | n=17  –0.24 ± 0.851 |
| End of Stage 2 | n=41  9.12 ± 0.740 | n=41  –0.25 ± 0.788 | n=8  9.46 ± 0.618 | n=8  –0.28 ± 0.829 |
| **Calcium-phosphorus product, mg^2^/dL^2^** |  |  |  |  |
| Baseline | n=65  61.5 ± 17.38 | — | n=19  65.9 ± 22.72 | — |
| End of Stage 1 | n=65  52.7 ± 17.27 | n=65  –8.0 ± 15.19 | n=16  63.1 ± 20.55 | n=16  –4.0 ± 27.58 |
| End of Stage 2 | n=40  48.5 ± 15.03 | n=40  –5.9 ± 17.33 | n=8  60.9 ± 16.00 | n=8  –1.5 ± 15.23 |
| **iPTH, pg/mL** |  |  |  |  |
| Baseline | n=65  286.42 ± 248.989 | — | n=19  350.17 ± 184.212 | — |
| End of Stage 1 | n=64  266.81 ± 197.847 | n=64  –23.48 ± 208.855 | n=15  433.01 ± 267.584 | n=15  54.00 ± 190.251 |
| End of Stage 2 | n=41  335.89 ± 310.403 | n=40  43.50 ± 366.585 | n=8  493.43 ± 340.967 | n=8  113.34 ± 256.138 |
| **Ferritin, µg/L** |  |  |  |  |
| Baseline | n=56  223.67 ± 274.071 | — | n=16  234.28 ± 228.825 | — |
| End of Stage 1 | n=58  322.59 ± 368.823 | n=51  48.59 ± 168.999 | n=15  310.46 ± 369.869 | n=14  59.27 ± 219.660 |
| End of Stage 2 | n=33  326.13 ± 304.864 | n=28  137.57 ± 149.176 | n=8  345.13 ± 366.230 | n=8  110.69 ± 211.054 |
| **Serum iron, µmol/L** |  |  |  |  |
| Baseline | n=65  13.20 ± 6.948 | — | n=19  15.25 ± 8.488 | — |
| End of Stage 1 | n=64  14.70 ± 7.425 | n=63  1.64 ± 8.946 | n=16  12.66 ± 4.821 | n=16  –0.18 (7.197) |
| End of Stage 2 | n=40  14.31 ± 6.005 | n=39  1.95 ± 6.940 | n=8  13.08 ± 4.967 | n=8  1.35 ± 9.691 |

CaAc, calcium acetate; iPTH, intact parathyroid hormone; SD, standard deviation; SFOH, sucroferric oxyhydroxide
